# Gender-Related Differences in the Dysfunctional Resting Networks of Migraine Suffers

**DOI:** 10.1371/journal.pone.0027049

**Published:** 2011-11-02

**Authors:** Jixin Liu, Wei Qin, Jiaofen Nan, Jing Li, Kai Yuan, Ling Zhao, Fang Zeng, Jinbo Sun, Dahua Yu, Minghao Dong, Peng Liu, Karen M. von Deneen, Qiyong Gong, Fanrong Liang, Jie Tian

**Affiliations:** 1 Life Sciences Research Center, School of Life Sciences and Technology, Xidian University, Xi'an, Shaanxi, China; 2 The 3rd Teaching Hospital, Chengdu University of Traditional Chinese Medicine, Chengdu, China; 3 Huaxi MR Research Center (HMRRC), Department of Radiology, The Center for Medical Imaging, West China Hospital of Sichuan University, Chengdu, Sichuan, People's Republic of China; 4 Institute of Automation, Chinese Academy of Sciences, Beijing, China; Institute of Psychology, Chinese Academy of Sciences, China

## Abstract

**Background:**

Migraine shows gender-specific incidence and has a higher prevalence in females. However, little is known about gender-related differences in dysfunctional brain organization, which may account for gender-specific vulnerability and characteristics of migraine. In this study, we considered gender-related differences in the topological property of resting functional networks.

**Methodology/Principal Findings:**

Data was obtained from 38 migraine patients (18 males and 20 females) and 38 healthy subjects (18 males and 20 females). We used the graph theory analysis, which becomes a powerful tool in investigating complex brain networks on a whole brain scale and could describe functional interactions between brain regions. Using this approach, we compared the brain functional networks between these two groups, and several network properties were investigated, such as small-worldness, network resilience, nodal centrality, and interregional connections. In our findings, these network characters were all disrupted in patients suffering from chronic migraine. More importantly, these functional damages in the migraine-affected brain had a skewed balance between males and females. In female patients, brain functional networks showed worse resilience, more regions exhibited decreased nodal centrality, and more functional connections revealed abnormalities than in male patients.

**Conclusions:**

These results indicated that migraine may have an additional influence on females and lead to more dysfunctional organization in their resting functional networks.

## Introduction

Migraine is a debilitating condition, characterized by severe headaches and nausea, which leads to disability, lost productivity, and a decreased overall quality of life [1]–[3]. Recently, researchers have shown an increased interest in the damage and dysregulation of the central nervous system (CNS) occurring in patients with headaches and migraine. In particular, Filippi et al. (2008), May (2009) and Chiapparini et al. (2010) provided summaries of the main investigations in the modern imaging field [4]–[6]. Based on neuroimaging findings, several brain regions in patients with migraine (PM) have been reported to show structural and functional alteration, including the insula (INS), anterior cingulate cortex (ACC), cerebellum, thalamus, prefrontal cortex (PFC), and temporal cortex [7]–[11]. These brain abnormalities were considered as the secondary effects of migraine, and may be the results of dysfunction from infrequent to frequent cortical overstimulation associated with headaches [4], [6]. Neuroimaging findings provide useful information on brain activity outside of headache attacks, and are just beginning to learn about CNS damage and cortical reorganization in migraine.

Recently, differences in the incidence of migraine between men and women have been observed [12], [13]. Stewart et al. (2008) pointed out that migraine had a cumulative lifetime incidence of 43% in women and 18% in men [12], which was about three times more common in women than in men [13]. As we know, gender is an important influential element in brain neural systems [14]. Anatomical and functional network organization in the human brain is associated with gender [15]–[17]. Yan et al. (2010) investigated gender differences in the structural brain networks and found that females had greater local efficiencies than males did [16]. Tian et al. (2011) demonstrated that there existed gender-related differences in the organization of brain functional networks [17]. Additionally, the gender difference is also an influential factor for behavior and cognitive performance [18], such as for the mental rotation test [19], emotion recognition test [20], and social sensitivity test [21]. However, to date, there has been little discussion on the cortical network organization differences between men and women with migraine. Due to the different incidence of this disease, there is the high possibility that migraine may have a different influence on the organization of cortical networks between males and females.

Previous studies focused on the local variation of regional cerebral blood flow (positron emission tomography, PET) and structural damage (voxel-based morphometry, VBM) [7], [11], [22], [23] in migraine. However, physiological effects of brain injury were best evaluated throughout all of the cortical networks, rather than assessed locally [24]. In this study, we used resting-state functional MRI to consider resting networks which can be conceived as neurocognitive entities that incorporate both local and global processes [25], [26]. Furthermore, differences in resting networks between patients and control subjects could imply differences in an underlying disease-specific brain abnormality [27]. The recent application of graph theory analysis (GTA) has become a powerful tool to investigate complex brain networks on a whole brain scale by defining a graph as a set of nodes (brain regions) and edges (functional connections) [28]–[30]. A graph represents the cortical network, where it describes the basis of cognitive processing for distributed functional interactions between brain regions [31]. Cortical network analysis may accelerate our understating of a dysregulated connectome in the PM.

Here, we investigated the topological properties of brain networks by using GTA and hypothesized that that there was a different connection model which arose from gender differences in PM. To test our aim, we constructed functional brain networks to characterize the interregional relationships between brain regions in PM and healthy controls (HC) respectively. We shed light on an integrative systems' perspective and detected the patients' abnormal network characteristics and their associations with gender.

## Materials and Methods

All research procedures were approved by the West China Hospital Subcommittee on Human Studies and were conducted in accordance with the Declaration of Helsinki.

### 2.1 Participants

Thirty-eight right-handed migraine patients (20 females, 32.3±8.7 years (mean age ± SD); 18 males, 33±7.9 years (mean age ± SD)) were recruited for the study (Table 1 and Table 2). All patients fulfilled the ICHD-II criteria for migraine without aura. According to Detsky et al. (2006), five questions were asked: (1) “Is it a pulsating headache?” (2) “Does it last between 4 and 72 hours without medication?” (3) “Is it unilateral?” (4) “Is there nausea?” and (5) “Is the headache disabling?” [32]. All patients answered “yes” to 4 or more of the 5 questions. In all patients, migraine attack frequency was 6.7±2.1 days/month, migraine attack duration was 14.6±7.1 hours, the migraine duration was 11.3±6.7 years, and the average pain intensity was 5.3±1.6 for the past four weeks. No patients had macroscopic brain T2-visible lesions on MRI scans. No patients had any history of drug abuse. All patients had been free from a typical migraine attack for at least 1 week prior to MRI examination. After scanning, all subjects reported that they did not experience any headaches or migraines during the experiment.

**Table 1:** Demographic characteristics of subjects.

Information	Healthy controls (n = 38)	Patients with migraine (n = 38)
Age (years)	32.6±6.9	32.5±8.2
Education (years)	11.7±4.8	12.0±3.4
Disease duration (years)	N/A	11.3±6.7
**Migraine attacks during past four weeks**		
Attack duration (hours)	N/A	14.6±7.1
Attack frequency (times)	N/A	6.7±2.1
Average pain intensity (0–10)	N/A	5.3±1.6

**Table 2:** Demographic characteristics of subjects.

Information	Healthy controls		Patients with migraine	
	Females (n = 20)	Males (n = 18)	Females (n = 20)	Males (n = 18)
Age (years)	31.1±9.3	33.3±2.1	32.3±8.7	33.0±7.9
Education (years)	12.2±6.1	11.5±3.4	12.1±4.3	11.8±2.8
**Migraine attacks during past four weeks**				
Disease duration (years)	N/A	N/A	10.9±7.4	8.1±2.3
Average duration of a migraine attack (hours)	N/A	N/A	12.9±7.7	14.5±4.4
Attack frequency (times)	N/A	N/A	4.0±1.9	4.4±2.1
Average pain intensity (0–10)	N/A	N/A	5.4±1.7	5.2±1.2

Thirty-eight age-, education- and gender-matched, healthy, right-handed controls (age 32.6±6.9 years) were recruited from the local community. All HC had no history of major medical illnesses, head trauma, and neuropsychiatric disorders, and had not used prescription medications within the last month. To exclude abnormal neurological findings, all HC were screened by a neurologist specialized in headaches. All subjects gave written, informed consent after the experimental procedures had been fully explained.

### 2.2 fMRI Data Acquisition

The fMRI experiments were carried out in a 3T GE scanner. We collected resting state scans of 205 continuous EPI functional volumes (TE 30 ms, TR 2 s, matrix 64×64, FOV 240 mm, flip angle 90°) for HC and patient groups. Subjects were instructed to keep their eyes closed, to not think about anything, and to stay awake during scanning. According to the subjects' reports, they all stayed awake during all scans. For spatial normalization and localization, a set of T1-weighted high-resolution anatomical images were acquired (TE 3.39 ms, TR 2.7 s, matrix 256×256, FOV 256 mm, flip angle 7°, in plane resolution 1×1 mm, slice thickness 1 mm).

### 2.3 Data preprocessing

Image preprocessing was carried out using SPM5 (http://www.fil.ion.ucl.ac.uk/spm). All datasets were initially corrected for temporal offsets using sinc interpolation and head movement-related effects using a six-parameter spatial transformation [33]. To minimize movement artifacts, individuals with an estimated maximum displacement in any direction larger than 1.5 mm or head rotation larger than 1.5° were discarded from the study. No data were excluded under this criterion. The mean EPI image for each subject was then co-registered to a corresponding T1-weighted high-resolution image volume and subsequently spatially normalized to the Montreal Neurological Institute (MNI) echoplanar imaging (EPI) template image and resampled to 2-mm isotropic voxels. In order to avoid local correlations, the spatially normalized data were not spatially smoothed in this study.

### 2.4 Construction of the cortical brain network

A key issue in characterizing the brain topological network is the construction of the functional connection matrix. In this study, automated anatomically labeled (AAL) template images were used [34], which divided the whole brain into 90 regions of interest (ROIs)(Table 3). These 90 brain regions were considered as a set of nodes in our network analysis. Then, the fMRI time series of all regions was filtered using a bandpass filter (0.01–0.08 Hz) to remove the effects of magnetic field drift and high-frequency noise. After that, the mean time courses from deep white matter, ventricles and the 6 rigid-body motion parameters were regressed out from the filtered time series. Finally, we computed the mean time series of each seed region and obtained a 90*90 matrix of the Pearson correlation coefficients between all possible connections of node pairs [35]–[37].

**Table 3:** Cortical and subcortical regions defined by the AAL template image in standard stereotaxic space.

Region	Abbreviation	Region	Abbreviation
Superior frontal gyrus, dorsolateral	SFGdor	Superior temporal gyrus	STG
Superior frontal gyrus, orbital	ORBsup	Superior temporal gyrus, temporal pole	TPOsup
Superior frontal gyrus, medial	SFGmed	Middle temporal gyrus	MTG
Superior frontal gyrus, medial orbital	ORBsupmed	Middle temporal gyrus, temporal pole	TPOmid
Middle frontal gyrus	MFG	Inferior temporal gyrus	ITG
Middle frontal gyrus, orbital	ORBmid	Heschl gyrus	HES
Inferior frontal gyrus, opercular	IFGoperc	Hippocampus	HIP
Inferior frontal gyrus, triangular	IFGtriang	Parahippocampal gyrus	PHG
Inferior frontal gyrus, orbital	ORBinf	Amygdala	AMYG
Gyrus rectus	REC	Insula	ANG
Anterior cingulate gyrus	ACG	Thalamus	THA
Olfactory cortex	OLF	Caudate nucleus	CAU
Superior parietal gyrus	SPL	Lenticular nucleus, putamen	PUT
Paracentral lobule	PCL	Lenticular nucleus, pallidum	PAL
Postcentral gyrus	PoCG	Calcarine fissure and surrounding cortex	CAL
Inferior parietal gyrus	IPL	Cuneus	CUN
Supramarginal gyrus	SMG	Lingual gyrus	LING
Angular gyrus	ANG	Superior occipital gyrus	SOG
Precuneus	PCUN	Middle occipital gyrus	MOG
Posterior cingulate gyrus	PCG	Inferior occipital gyrus	IOG
Precentral gyrus	PreCG	Fusiform gyrus	FFG
Supplementary motor area	SMA	Rolandic operculum	ROL
Median- and para-cingulate gyrus	MCG		

As mentioned in previous studies [36]–[38], we invested the brain's topological properties by way of binarized graphs in which each correlation matrix was thresholded and converted to the adjacency matrix. The adjacency matrix is a means of representing which nodes of the graph are adjacent to other vertices with 1 dedicating the existing edge and 0 indicating the absence of an edge between the two nodes. A brain functional connection could be represented as an undirected edge if the correlation coefficient between the two nodes is larger than a correlation threshold corresponding to a sparsity value which was defined as the total number of edges in a network divided by the maximum possible number of edges [39]. In this way, the resulting graphs would be comprised of the same number of connections, which make cortical networks in the HC and patient groups to have the same wiring cost [39], [40]. We thresholded each correlation matrix repeatedly over a wide range of thresholds (15% to 25%) and then estimated the network properties at each threshold value. To consider the abnormal connectivity and cortical hubs between the HC and patient groups, the network pattern at threshold sparsity (S)  = 15% was shown as being typical in the following analysis. This value was the lowest threshold that could avoid generating isolate node in the networks (the cortical networks are fully connected), and it could also minimize the number of spurious edges in the networks [41], [42]. This threshold was employed in several previous GTA studies [15]–[17], [40], [43], [44].

### 2.5 Small-world properties analysis

The discovery of the small-world network, in which most nodes are not neighbors of one another but can be attained via a small number of steps, has offered new insights into various aspects of the network properties [45], [46]. Small-world structure, which has a highly efficient neuronal architecture, was frequently found in the brain network organization [38], [41]. Several network metrics were calculated to assess small-world properties. The key parameters of a small-world network were the clustering coefficient of a network C and the mean minimum path length L. The clustering coefficient of a node 0<Ci<1 is a ratio that defines the proportion of possible connections that actually exist between the nearest neighbors of a node [36], [37], [45]. The clustering coefficient of a network C is the average over each node's clustering coefficient:
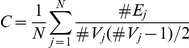



where N is the total number of nodes in the network, 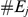 is the number of edges connecting the neighbors of node j, and 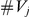 is the number of neighbors of node j. The minimum path length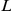 is the average of the shortest path lengths over each possible pair of vertices [36], [37], [45]:
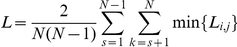



where 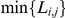 is the shortest path length between the 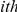 node and the 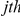 node, and the path length is defined as the number of edges included in the path. Corresponding parameters for a random graph of C and L with the same number of nodes were also calculated, as denoted by 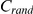 and 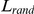. The original degree distribution is preserved when random networks are created with a random reconnection of the edges in the network of origin at least ten times to destroy the neighboring structures [47]. Random networks have a small average shortest path length, but with limited local interconnections resulting in a small 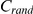 and 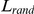. A graph is considered small-world if its average clustering coefficient C is significantly higher than a random graph constructed on the same number of nodes, and if the graph has a small average shortest path length [36]. We examined the ratio 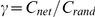 and the ratio 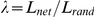 in our resting networks. The ratio 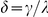 could be summarized for small-world networks as typically being >1 [36].

### Betweenness centrality

Based on anatomical evidence, Hagmann et al. (2008) pointed out that the brain region within the structural core was composed of connector hubs that linked all of the major structural modules [48]. These cortical hubs play a critical role in integrating diverse informational sources and balancing the opposing pressure to evolve segregated, specialized pathways [49]. To calculate the relative importance of a vertex in a graph, we applied the betweenness centrality measurement in our analysis. Nodes that occur on many shortest paths between other nodes have higher betweenness than those that do not [50]:
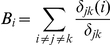



where 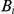 is the betweenness centrality of a node *i,* 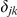 is the shortest path number from node *j* to node *k*, and 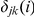 is the shortest path number from node *j* to node *k* that passes through node *i*. 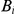 estimates the significance of a brain region over information flow between other brain regions in the cortical networks.

### 2.6 Network robustness analysis

To assess the resilience of the brain networks to targeted attack in the pathological conditions of a migraine, we used the approach described by Albert and Barabasi (2002) [51]. Targeted attack of the network was stimulated by selecting the node with the largest betweenness centrality. We then removed it and assessed the size of the remaining largest connected cluster. We repeated this process and incrementally eliminated additional nodes in decreasing order of their network betweenness centrality across all subjects [36], [42], [51]. Finally, we obtained network robustness measures in the HC and PM group respectively and estimated their differences.

### 2.7 Interregional correlations analysis

While graph visualization provided strong hints of gender-related dysregulated brain regions in PM, we further evaluated the abnormal interregional correlations of the resting networks in the male and female PM. The correlation coefficient between the two regions was preserved as the weights of the edges. Then, Fisher's z transformation was performed to convert the correlation coefficients to a z value [40].

### 2.8 Statistical analysis

To investigate whether there were significant differences in the topological properties of the resting networks between HC and PM, a two-way ANOVA was performed on each network metric (small-worldness, betweenness centrality, network robustness and interregional correlations) to model gender (male vs. female) and disease state (patients vs. controls) effects simultaneously. The test evaluated the hypothesis that gender, disease state and interaction effects were all the same against the alternative that they were not all the same. We paid more attention to the significant interactions between gender and disease state, and performed a test to determine which pairs of effects were significantly different. In addition, we performed partial correlation analyses between the changes of network organization and migraine duration across all subjects while controlling for age.

## Results

### 3.1 Small-world property

**Figure 1:**
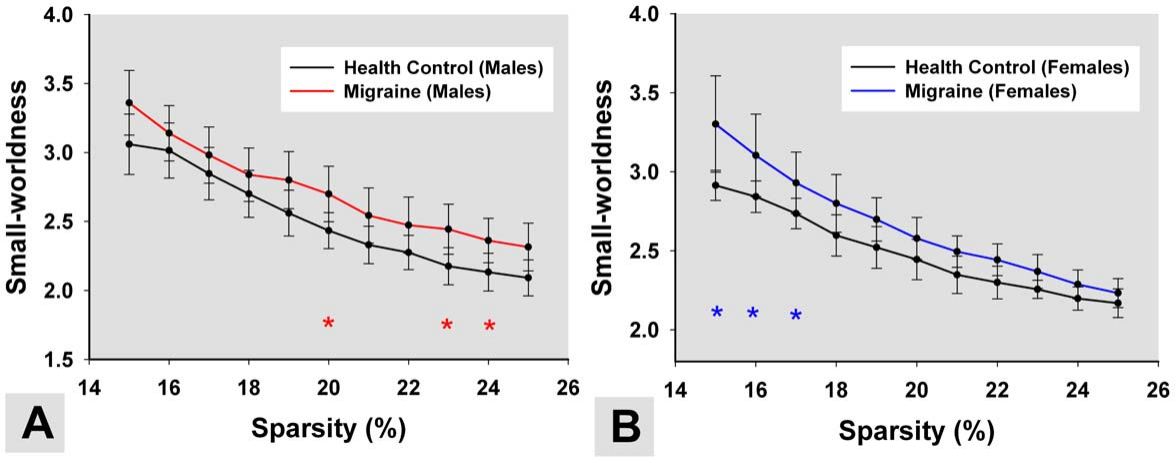
The mean and standard deviation of small-worldness from the resting networks at different sparsity values. The colored lines show small-worldness differences among (A) male PM, (B) female PM and matched controls. The horizontal stars indicate the significant difference of the small-worldness between HC and PM (*p*<0.05, FDR corrected).

In the current study, we constructed resting functional networks by using GTA between gender (male vs. female) and disease state (patients vs. controls). Small-world network properties were obtained at different network sparsity values (Fig. 1 A and B) from 15% to 25% in 0.01 increments. For the significant interactions, we used a multiple comparison test to determine which pairs of effects were significantly different. For males, small-world network organization was significant altered at 20%, 23% and 24% between PM and HC (Fig. 1 A, *p*<0.05, FDR corrected). For females, there were significant small-worldness differences to the migraine compared to HC at 15%, 16% and 17% (Fig. 1 B, *p*<0.05, FDR corrected). The horizontal star line in Fig.1 indicates the significant difference in the network parameters between the HC and PM groups among all of the thresholds.

**Figure 2:**
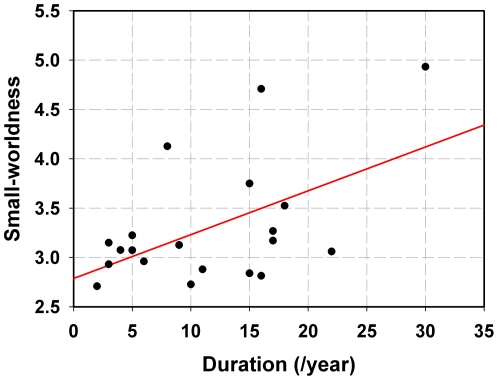
Correlation between the small-worldness of female PM resting networks and the migraine duration while controlling for patients' age at threshold S = 15%.

The sparsity S = 15% was selected as being typical in the following network analysis in all subjects (see Materials and Methods). The results exhibited that the small-worldness in female PM was positively correlated with the duration of migraine by applying a linear partial correlation model controlling for the patients' age (Fig. 2, r = 0.53, *p* = 0.02). We did not find any significant correlation in male PM (*p*>0.05).

### 3.2 Network robustness

**Figure 3:**
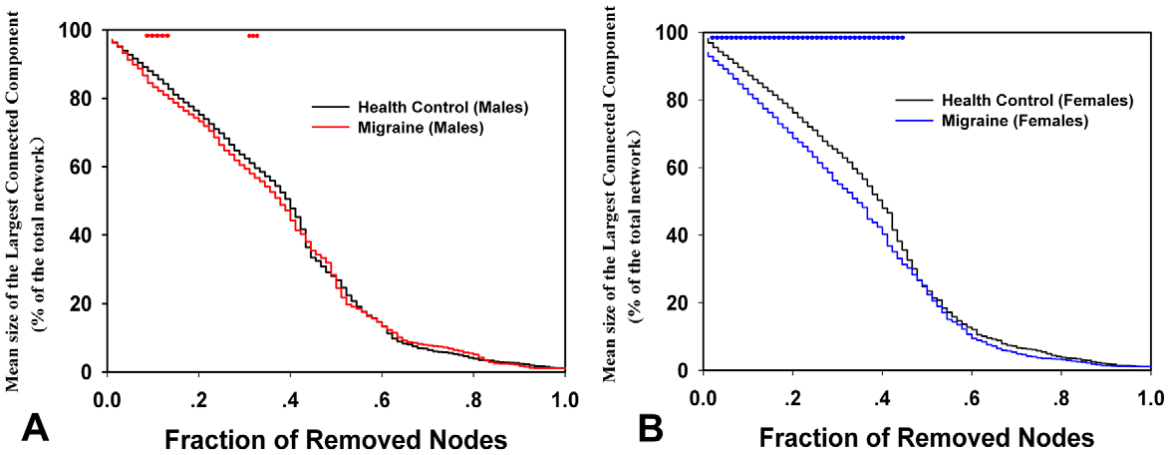
Network robustness analysis for targeted attacks. Graph presents the mean size of the largest connected component versus the proportion of total nodes eliminated by targeted attacks among (A) male PM, (B) female PM and matched controls. The horizontal dotted line indicates the significant difference between HC and PM (p<0.05).

The network robustness evaluation could provide us with a precise quantitative measure of global impairment for PM. Our results showed that the resting networks in HC were resilient to targeted attack than in PM (Fig.3). Specifically, the mean size of the largest connected component in the resting networks of male HC was decreased to 60% by targeted attack when the top 33% nodes with high betweenness values were removed, but was 30% of the nodes in male PM (Fig. 3 A). For females, the brain networks in HC were disintegrated to the same extent when the top 34% nodes were deleted, but was 27% of the nodes in female PM (Fig. 3 B). Comparing topological properties of the male PM resting network to that of the female PM, gender-related differences were found in our results. As can be seen from Figure 3, the network robustness revealed different degrees of loss in male and female PM. Significant network breakdowns (*p*<0.05) were found in male and female PM due to targeted node attacks (horizontal dotted line in Fig.3).

### 3.3 Nodal centrality

**Figure 4:**
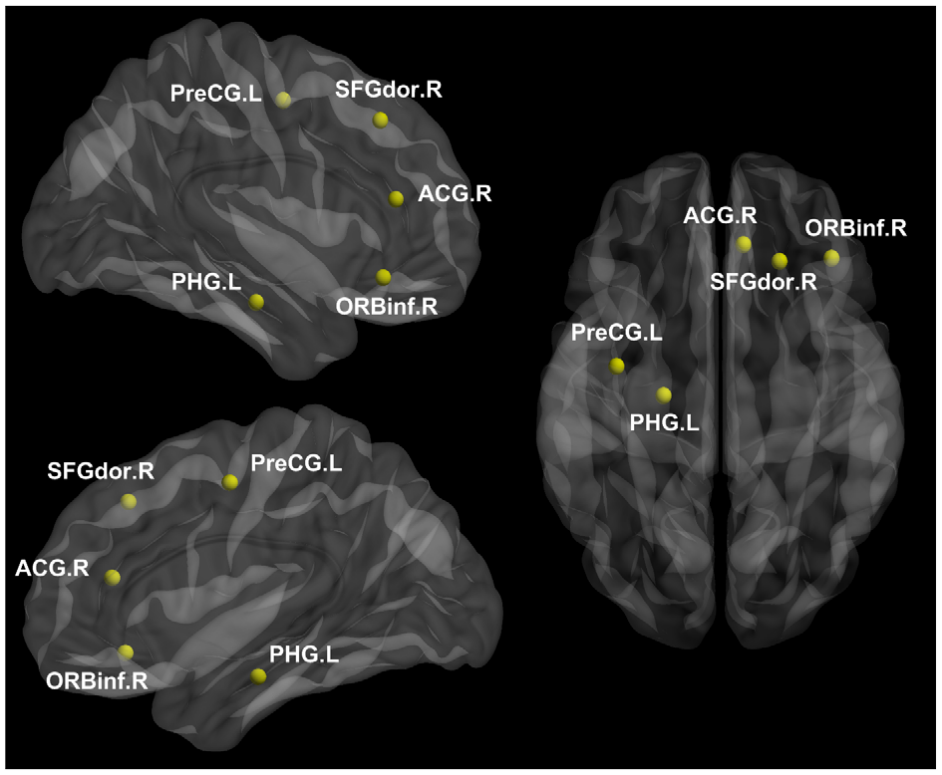
Significant interactions (nodal centrality differences) between gender (male vs. female) and disease state (patients vs. controls) effects. A two-way ANOVA was performed (p<0.05, corrected). This figure was visualized with the BrainNet viewer.

Based on each nodal betweenness centrality, our aim was to assess the prominent nodal centrality differences between the HC and PM resting networks, and investigate the gender-related differences in local impairments between male and female PM networks. Five ROIs showed a significant interaction between gender (male vs. female) and disease state (patients vs. controls) from the whole brain ANOVA analysis (Fig. 4, *p*<0.05, FDR corrected). As shown in Figure 4, these brain regions included the precentral gyrus (PreCG), dorsolateral superior frontal gyrus (SFGdor), orbital inferior frontal gyrus (ORBinf), anterior cingulate gyrus (ACG), and parahippocampal gyrus (PHG). Furthermore, there was a significant decrease in PM females compared to HC in all of these brain regions (Table 4). In male groups, only the ORBinf exhibited a significant decrease in nodal centrality in PM as compared to HC. In addition, the nodal centrality of the PreCG and ACG presented a significant correlation with the duration of migraine (Table 4) in female PM.

**Table 4:** The nodal centrality (betweeness) across gender and disease state.

Regions	Classification	*males*		*corrected p (HC vs. PM)*	*correlation with migraine duration*		*females*		*corrected p (HC vs. PM)*	*correlation with migraine duration*	
		*HC*	*PM*				*HC*	*PM*			
		mean ± sd	mean ± sd	value	r	p	mean ± sd	mean ± sd	value	r	p
PreCG.L	Primary	160.1±180.2	99.1±67.3	>0.05	−0.34	>0.05	227.7±247.2	107.4±83.3	**<0.05**	−0.51	**0.02**
SFGdor.R	Association	166.0±154.9	168.5±82	>0.05	−0.21	>0.05	261.3±122.8	152.4±114	**<0.05**	−0.17	>0.05
ORBinf.R	Paralimbic	478.7±88.1	54.5±38	**<0.05**	−0.07	>0.05	198.9±142.3	116.8±92.5	**<0.05**	−0.28	>0.05
ACG.R	Paralimbic	43.6±55.9	55.1±44	>0.05	−0.31	>0.05	95.6±98.0	38.4±49.7	**<0.05**	−0.52	**0.02**
PHG.L	Paralimbic	2.1±3.0	66.5 ±103.5	>0.05	−0.12	>0.05	167.6±181.6	43.4±72.1	**<0.05**	0.06	>0.05

### 3.4 Interregional correlations

**Figure 5:**
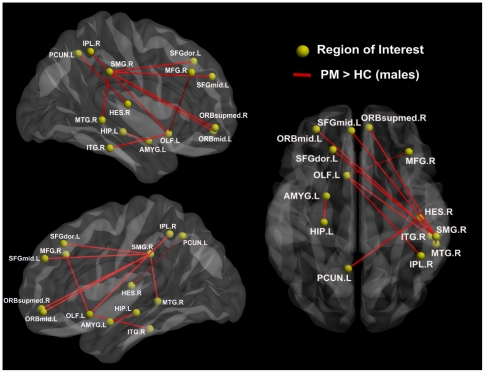
Significant differences in the intensity of the functional connection in male PM resting networks. Ten connections (red lines) showed increased intensity in the male PM resting networks (migraine > HC, p<0.05, FDR corrected). This figure was visualized with the BrainNet viewer.

**Figure 6:**
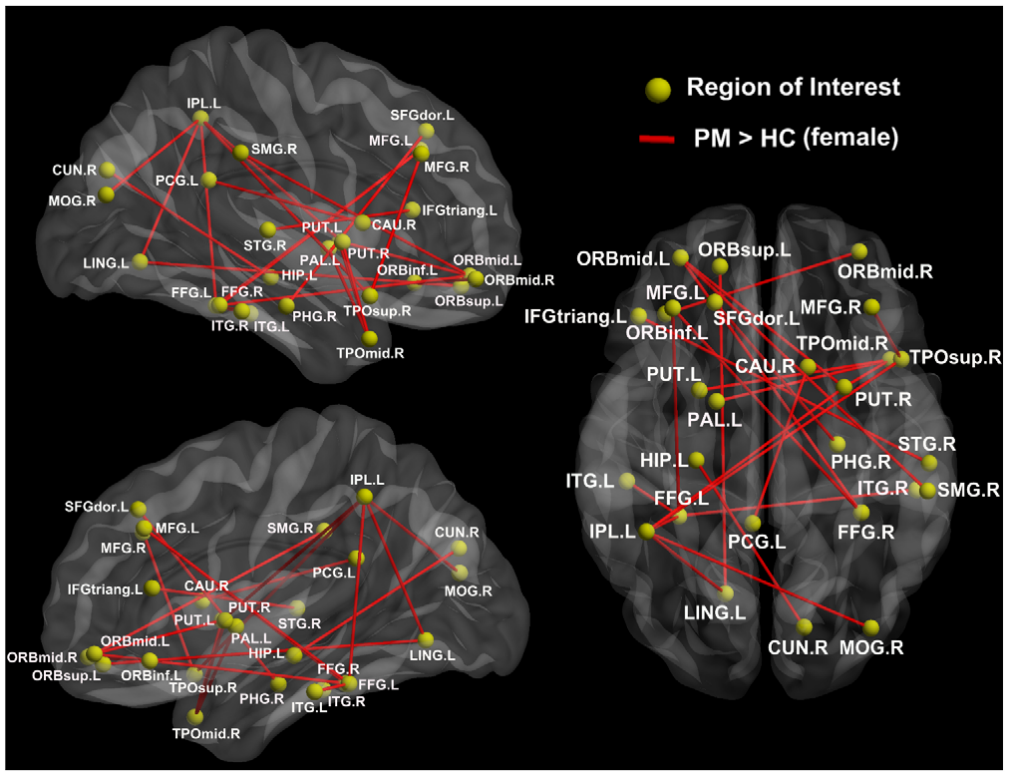
Significant differences in the intensity of the functional connections in the female PM resting networks. Ten connections (red lines) showed increased intensity in the female PM resting networks (migraine > HC, p<0.05, FDR corrected). This figure was visualized with the BrainNet viewer.

Several pairs of connections were significantly altered in PM (*p*<0.05, FDR corrected) (Fig. 5 and Fig. 6). In the male PM group, significantly increased connections were found in the PFC, SMG, amygdala (AMYG), HIP, inferior parietal lobule and temporal lobes (Fig. 5). In the female PM group, the network of patients was more disorganized compared with matched controls, and more regions showed significant increases in interregional correlations (Fig. 6), including the orbital part of the PFC, posterior cingulate gyrus (PCG), PHG, cuneus (CUN), putamen (PUT), caudate (CAU), parietal lobule, temporal lobes, and occipital cortex (Fig. 6). No significantly decreased connections were found in PM.

## Discussion

In this study, we viewed resting functional networks of migraine from an integration perspective. Compared with HC, we found abnormal topological organization in PM brain networks, including the small-world property, resilience, nodal centrality, and interregional connections. Furthermore, gender-related differences of these network parameters in migraine patients were also observed.

Small-world models are useful for connectivity studies of nervous systems because they have high clustering and a short path length which confers the capability for both specialized or modular processing in local neighborhoods, as well as including distributed or integrated processing over the entire network [36]. In the present study, salient small-worldness was found in the resting networks of HC, consistent with several previous cortical network studies [52]. However, both male and female PM resting networks showed different small-world scalars over a wide range of sparsity values as compared with HC, demonstrating that more variable topological properties existed in these two groups (Fig. 1). Previous studies demonstrated that a migraine was associated with a significant gray matter reduction in several brain areas [23], [53]–[56]. While subtle brain lesions led to abnormal brain function [57]–[59], long-term headaches could alter functional brain organization of migraine patients [60]. As mentioned above, our results indicated that the small-world property was disrupted in PM, which may be the consequence of frequent migraine-related nociceptive input in patients' daily lives.

The small-worldness measurement of the resting network for HC, male and female PM could provide valuable insights into the characteristics of abnormal organization in a migraine patient's brain. It still needs further analysis to conclude the global damage for the resting network in migraine and its gender-related differences. Achard et al. (2006) suggested that healthy brain organization had advantages in terms of robustness to a selective attack on cortical hubs, and it could confer resilience against the loss of network functionality in the face of a pathological attack [36]. As compared with HC, male and female PM resting networks tended to fragment rapidly when responding to targeted attacks (Fig. 3). These findings might be interpreted as the results of long-term and high-frequency headache attacks, which give rise to lesions and to migraine-related loss of functional integrity, thus leading to unfavorable influences on the functional connections within the resting functional networks. Interestingly, we noted differences between male and female PM in their network robustness. Resting networks in female PM exhibited the worst tolerance against targeted node removal among the three groups. This indicated that the functional networks in females may be more vulnerable to disruption of the developmental aberration resulting from migraine.

Many studies have investigated the central pain network involved in nociceptive processing in the human brain, and several cortical and subcortical brain regions exhibited altered neural activity in response to pain in different experimental conditions [23], [61], [62]. In our study, there was a significant nodal centrality decrease in the PreCG, SFGdor, ORBinf, ACG, and PHG (Fig. 4 and Table 4). These brain regions were largely considered to be involved in pain processing [63]. Our results were consistent with many previous studies of migraine on brain structure and function [22], [23], [54], [63], [64]. Furthermore, we found differences between male and female PM in the distribution of abnormal nodal centrality as compared to matched controls (Table 4). While male PM nodal centrality only decreased in the orbital part of the PFC, more regions showed less nodal centrality in female PM. From our observations, it could be inferred that migraine may affect males and females differently in the organizational patterns of brain functional networks in PM. Moreover, nodal centrality is a useful measure for the relative importance of a brain region to integrate diverse global information into the resting networks [16], [17], [40]. More regions showed significantly decreased centrality in females indicating more brain damage done in female PM, which may have a strong possibility of resulting in abnormal information integration during the experience and the anticipation of pain [6].

Figure 5 and Figure 6 present abnormal interregional correlations in male and female PM. Compared with matched controls, there were many functional connections showing a significant increase in the intensity of the interregional correlations, mainly in the SI, SII, SMG, PFC, striatum, HIP, PHG, AMYG, occipital cortices, and temporal cortices. During noxious stimuli, most of these regions were significantly activated to mediate the unpleasant-affective dimension of pain and the motivation to escape from noxious events in central pain processing [4], [65]–[67]. According to previous studies [4]–[6], these dysfunctional interregional correlations observed between brain regions within the pain processing networks could be understood as a brain injury and may be the result of the secondary effect of having migraine. It is important to note that abnormal functional connectivities in the chronically migraine-inflicted brain have a skewed balance between males and females. More dysfunctional connections were found in the female PM resting networks (Fig. 6). This finding was consistent with our results above that migraine had a distinction between male and female patients, where the resting networks of female PM had more functional abnormalities than in males. Recently, Kruit et al. (2004) pointed out that women, but not men, with migraine were associated with increased risk of white matter abnormalities [68]. There exist gender differences in the underlying brain network organization [15]–[17], [69]. Studies showed that white matter in women was more efficiently utilized [15], [70]. Gong et al. (2009) utilized diffusion tensor imaging tractography to construct cortical structure networks, and that the cortical networks of women had both higher overall global and local efficiency [15]. Gur et al. (1999) found a stronger association between white matter volume and cognitive performance in women [70]. According to our results, this may have a potential correlation between migraine and female brain characteristics. It is possible that the distinct features of female cortical networks may likely suffer more brain damage in females with migraine. We inferred that women's cortical networks may have a higher risk for migraine, which may explain the different prevalence of migraine between males and females.

There is a methodological limitation to the present study. While a graph represents functional connection relationships between brain regions, GTA characterizes the functional brain network by constructing a voxel-based network with each voxel as a network node [47], [49], or by constructing a region-based network with nodes corresponding to anatomically-defined ROIs [71]. Several studies also pointed out that different parcellation strategies of graph analytical techniques may result in a considerable variation in the exact values defining key parameters of network organization [72], [73]. In our study, functional brain networks were constructed at a regional level by dividing the brain into 90 regions. We do not know if the dysfunctional network organization of PM we found will change significantly with different parcellation strategies. To test the reproducibility of our results, future studies needs to consider the effect that a specific parcellation approach has on graph analytical findings.

In summary, by exploring the topological properties of the resting brain, we investigated the brain organization of migraine that included a gender component. We observed that functional damages showed a skewed balance between males and females in their network robustness, nodal centrality, and functional connections. The resting networks of females were more vulnerable to migraine. Our findings may help us better understand gender differences in PM during the resting state. Gender-related differences should be considered when designing experiments or interpreting results in future migraine studies.
